# Language Preferences on Websites and in Google Searches for Human Health and Food Information

**DOI:** 10.2196/jmir.9.2.e18

**Published:** 2007-06-28

**Authors:** Punam Mony Singh, Carly A Wight, Olcan Sercinoglu, David C Wilson, Artem Boytsov, Manish N Raizada

**Affiliations:** ^5^Croplink Global Food Security InitiativeDepartment of Plant AgricultureUniversity of GuelphGuelphONCanada; ^4^Department of PsychologyUniversity of GuelphGuelphONCanada; ^3^Google IncMountain ViewCAUSA; ^2^Croplink Global Food Security InitiativeDepartment of Plant AgricultureUniversity of GuelphGuelphONCanada; ^1^Respirology ProgramFaculty of MedicineUniversity of TorontoTorontoONCanada

**Keywords:** Health, Internet, Google, language, indigenous, food security, immigrant, avian flu, tuberculosis, maize, schizophrenia, nutrition, linguistic

## Abstract

**Background:**

While it is known that the majority of pages on the World Wide Web are in English, little is known about the preferred language of users searching for health information online.

**Objectives:**

(1) To help global and domestic publishers, for example health and food agencies, to determine the need for translation of online information from English into local languages. (2) To help these agencies determine which language(s) they should select when publishing information online in target nations and for target subpopulations within nations.

**Methods:**

To estimate the percentage of Web publishers that translate their health and food websites, we measured the frequency at which domain names retrieved by Google overlap for language translations of the same health-related search term. To quantify language choice of searchers from different countries, Google provided estimates of the rate at which its search engine was queried in six languages relative to English for the terms “avian flu,” “tuberculosis,” “schizophrenia,” and “maize” (corn) from January 2004 to April 2006. The estimate was based on a 20% sample of all Google queries from 227 nations.

**Results:**

We estimate that 80%-90% of health- and food-related institutions do not translate their websites into multiple languages, even when the information concerns pandemic disease such as avian influenza. Although Internet users are often well-educated, there was a strong preference for searching for health and food information in the local language, rather than English. For “avian flu,” we found that only 1% of searches in non-English-speaking nations were in English, whereas for “tuberculosis” or “schizophrenia,” about 4%-40% of searches in non-English countries employed English. A subset of searches for health information presumably originating from immigrants occurred in their native tongue, not the language of the adopted country. However, Spanish-language online searches for “avian flu,” “schizophrenia,” and “maize/corn” in the United States occurred at only <1% of the English search rate, although the US online Hispanic population constitutes 12% of the total US online population. Sub-Saharan Africa and Bangladesh searches for health information occurred in unexpected languages, perhaps reflecting the presence of aid workers and the global migration of Internet users, respectively. In Latin America, indigenous-language search terms were often used rather than Spanish.

**Conclusions:**

(1) Based on the strong preference for searching the Internet for health information in the local language, indigenous language, or immigrant language of origin, global and domestic health and food agencies should continue their efforts to translate their institutional websites into more languages. (2) We have provided linguistic online search pattern data to help health and food agencies better select languages for targeted website publishing.

## Introduction

The World Wide Web has more than 15 billion pages [1] and has become an important source of health-related information [2-5]. In the United States, for example, it has been reported that 82% of female users and 77% of male users used the Internet to obtain medical information on a routine basis [6]. Google searches have even been shown to assist physicians in the correct diagnosis of medical ailments [7]. However, two thirds of the pages on the Web are published in English [8,9], even though the world has over 5 billion non-English speakers [10], including approximately 700 million non-English-speaking Internet users [8]. In fact, the vast majority of the world’s 6900 living linguistic groups [10] have little Web content available in their language [8,9]. Adding to this problem, search engines such as Google do not translate search terms into other languages [9]—perhaps a surprise to many users. To overcome linguistic barriers, the World Health Organization (WHO) and the Food and Agricultural Organization (FAO) now publish their websites in six and four major languages, respectively. However, other globally authoritative organizations, such as the Centers for Disease Control (CDC) in the United States, primarily publish online information in only one or two languages of domestic importance.

In spite of the significant challenges created by linguistic differences in effectively communicating health information to the world’s peoples online, we could find little quantitative data on this issue. Do the world’s online users, presumably wealthier and more educated than the general population, primarily search online for health information in their local language, or do they employ Web-prevalent languages such as English? Are current online translation efforts by the world’s health and food agencies beneficial, and should these agencies be spending more resources on these efforts? In a world of human migration, which language(s) should domestic governments and international agencies use in order to communicate online health information to target populations? To transmit information to front-line health professionals in developing nations, a group that can include international aid workers from wealthy nations, which language(s) should be employed to target a particular nation? Do indigenous peoples search for online health-related information using search terms belonging to their own language or the colonial language? Real-time, accurate communication of health information might be especially critical during a pandemic infectious disease outbreak or famine. To begin to answer these questions, we have used a case-study approach that examines linguistic preferences in Internet search engine queries.

Specifically, we measured search patterns on Google for four health- and food-related terms in seven languages in 227 nations. The four search terms we chose for our study were “avian flu,” “tuberculosis,” “schizophrenia,” and “maize” (corn). We chose “avian flu” because it is an ideal model for searching for online information concerning an emerging infectious disease pandemic; as of August 2006, avian influenza (virus subtype H5N1) had killed 141 people in 10 countries [11,12] in addition to prompting the slaughter of millions of animals [13]. We chose “tuberculosis” because it is a good model for searching an established global infectious disease, as it is a major cause of death for HIV-positive patients [14] and currently afflicts about 15 million people in 207 nations [15]. We chose “schizophrenia” because psychiatric and neurological diseases affect more than 450 million people globally [16,17] and because online mental health information has the potential to help nations that have few mental health specialists [17]. Schizophrenia afflicts over 24 million people worldwide [18]. Finally, because there are currently around 850 million chronically undernourished people in the world [19], under frequent threat of famine, and because malnutrition is a major underlying contributor to infectious disease susceptibility [20], we chose “maize” (corn) because it is an important search term for global food security agencies. Maize supplies one third of all human calories in Latin America and Sub-Saharan Africa [19] and, combined with its genetic relatives rice and wheat, supplies approximately 50% of all human calories globally, either directly or via animal feed [19].

In this paper, we quantitatively demonstrate the need for health and food website translation that not only targets the world’s major languages, but also linguistic minorities within nations, including immigrants, foreign aid workers, and indigenous groups.

## Methods

### Measuring the Extent to Which Health and Food Agencies Translate Websites

When an online publisher translates its Web site across languages, the same source domain name can be found in the different result lists of a search engine when entering different translated search terms. In an URL, the domain name is the identifier before the first slash (eg, for the WHO, the domain name is www.who.int). Therefore, to estimate the percentage of institutions, news agencies, and other sources that translate their health and food websites, we measured the frequency that domain names overlap (institutional sources overlap) in different results lists when Google is queried using different language translations of the same search term (eg, English “tuberculosis” versus Bahasa Indonesia “tuberkulosa”). The language translations used in this study are shown in Multimedia Appendix 1. We first extracted URLs from Google.com listed as “the most relevant” (about 500-1000 URLs) based on the Google page rank algorithm [21] on July 10, 2006. We then excluded nonunique domain names from within each query group; the remaining unique subset was then compared to the URLs retrieved using the comparison language.

### Measurements of Online Search Rates and Language Choice

For each of the four terms and their translations, we measured the rate at which users from 227 different countries searched Google (Multimedia Appendix 2). The country of origin for each search was identified using the geographic locations of Internet Protocol (IP) addresses. The search rates were based on proprietary Google Inc. (Mountain View, CA) data using an algorithm that measured 20% of all first-page Web search queries from January 2004 to April 2006.

The search rate, *M*, for a term, *T*, originating from a specific country was then estimated from this large sample: the algorithm calculated the ratio of searches using a specific term (*T*) divided by all searches (*A*) from that nation during the time period, such that *M* = *T*/*A*. Multimedia Appendix 2 contains the standard error (SE) for each search rate. To calculate the standard error, a normal distribution for the statistic was assumed. To convert standard error to the 95% confidence interval of the estimate, the following formula was used: 95% CI = *M* ± (1.96 × SE). Only the search rates for selected languages are shown for each nation, not the total search rate across languages or the total number of searches, which are proprietary.

For ease of database searching and validation, we chose languages that share a Latin-based script similar to English. For detailed analysis, the languages chosen were English, Bahasa Indonesia, Spanish, Portuguese, German, and French, as they represent the primary or secondary language of about 100-500 million people worldwide and/or are significant post-colonial languages [10]. Because Turkey has reported cases of avian influenza [11,12], Turkish was added as a language of interest. A search term may be common to multiple languages, not all of which are noted here. Multimedia Appendix 1 contains a complete list of the language translations used to retrieve search rate data from the Google database.

### Health and Food Security Indicators

For the number of human avian influenza cases (subtype H5N1), we employed the WHO Epidemic and Pandemic Alert and Response Database [12], updated on August 9, 2006. For the number of poultry outbreaks of type H5 since 2003, we used the August 16, 2006 Avian Flu Update from the Organisation Mondiale de la Santé Animale (OiE) [World Organisation for Animal Health] [13]. For tuberculosis (TB), we used Millennium Development Goal (MDG) Indicator 23, the estimated TB prevalence in 2004 from the Global Tuberculosis Database of the WHO Global Health Atlas [15]. The number of psychiatrists per 100000 people in 2005 was obtained from Project Atlas: Resources for Mental Health and Neurological Disorders, from the WHO Global Health Atlas Database [17]. The most recent maize consumption data (kcal/person/day in 2004) was from the Supply Utilization Accounts (SUA) Database of the Food and Agricultural Organization Statistical Division (FAOSTAT) [19].

## Results

### Measuring the Extent to Which Institutions Currently Translate Online Information

We measured the extent to which the world’s institutions, including health and food agencies, news organizations, and other sources, currently publish Web content in multiple languages. To quantify this, we measured the institutional source (host-domain) overlap between search results (first 500-1000 URLs) retrieved using English-language queries versus select comparison languages (Table 1). For example, as shown in Table 1, “avian flu” resulted in 906 hits, which included 539 unique hosts. The same search in French resulted in 801 hits, which included 375 hosts. Only 7.1% of the 539 hosts found in the English search were also found in the French search, while 10.1% of the 375 hosts found with the French search were also found in the English search. This means that approximately 7.1%-10.1% of all hosts have an English/French translation.

For “avian flu,” which has afflicted 56 people in Indonesia [12], only 6.8% and 2.4% of institutional domain names overlapped between Bahasa Indonesia searches and English-language searches, respectively (Table 1). For “tuberculosis,” we discovered only about 9% overlap in the institutional domain names retrieved for English and French/Portuguese translations: French and Portuguese are widely spoken by TB-afflicted nations in Africa and Brazil [15]. The host domain overlap was typically less than 10% between European languages; we were surprised by this low rate given the presence of common governmental institutions in Europe (eg, European Union). We extrapolate that 80%-90% of health- and food-related institutions do not translate their websites into multiple languages, even when the information concerns pandemic disease. This does not exclude the possibility that other agencies, such as domestic health agencies, might be translating this information.

We then specifically screened for Web pages belonging to the WHO and the CDC, both authoritative agencies for infectious disease information. Because the first page of search results is the most viewed [22], the rank order in which search results appear is critical. When we searched for “avian flu” in English on Google.com, the CDC website was the first hit, followed second by the WHO website. In the French translation, however, no CDC-affiliated Web pages appeared in the first 100 hits. When we searched Google Indonesia using the Bahasa Indonesia translation of “avian flu,” a WHO-affiliated Web page did not appear until page three of the search results (rank 21), and the first CDC-affiliated page appeared on page six (rank 63). Most significant, by searching for “avian flu” in Turkish using Google Turkey, we were unable to retrieve the websites of either the WHO or CDC in the first 500 search results.

As a cautionary note, we found that a single accent or special character in the search term sometimes changed the rank order of health search results significantly, consistent with more detailed analysis conducted for nonmedical terms [9,23]: for example, for avian flu, we found that “gripe aviária” (accent, Portuguese) yielded 49/100 top search results of Brazilian origin (ie, “br” in domain name), whereas “gripe aviaria” (no accent, Portuguese) yielded 40/100 top search results from Spanish-speaking nations and only 4/100 search results from Brazil. This is significant, because we found that Brazil, Portugal, and other Portuguese-speaking nations (Angola, Mozambique) searched Google for this term with and without the accent at nearly equal rates (Multimedia Appendix 2).

**Table 1:** Overlap in the institutional domain names retrieved by Google to measure the extent to which institutions translate websites across languages

**English** **Search** **Term**	**Comparison** **Language**	**English Language Search**			**Comparative Language Search**		
		**URLs**	**Unique** **Hosts**	**Host Overlap with** **Comparison Languages**	**URLs**	**Unique** **Hosts**	**Host Overlap with** **English**
							
Avian flu	French	906	539	7.1%	801	375	10.1%
Avian flu	Indonesian	906	539	2.4%	472	190	6.8%
Tuberculosis	French Portuguese Dutch	834	473	8.5%	790	426	9.4%
Tuberculosis	German Danish Afrikaans	834	473	0.0%	809	443	0.0%
Tuberculosis	Bahasa Indonesia	834	473	0.8%	462	273	1.5%
Schizophrenia	Spanish Portuguese	813	444	10.8%	764	518	9.3%
Schizophrenia	Bahasa Indonesia Malay (Bahasa Malayu)	813	444	17.1%	748	463	16.4%
Maize	Spanish	828	421	7.8%	830	543	6.1%

### Language-Specific Searching of Infectious Disease Information

Though we did not retrieve many WHO- and CDC-affiliated Web pages when we searched across different languages, one possibility is that Internet users, many of whom are well-educated, are supplementing their Google searches for online health information by searching in English. If true, then there would be less of a need for the WHO and other global agencies to translate online information into diverse languages.

In our case study, we found the actual results to be variable (Table 2; Multimedia Appendix 2): for “avian flu,” we often found that only 1% of searches in non-English-speaking nations were in English, whereas for “tuberculosis” or “schizophrenia,” about 4%-40% of searches in non-English countries employed English. Brazil, which had an estimated 141000 cases of TB in 2004 [15], had an 18-fold higher query rate for “tuberculose” (Portuguese) than “tuberculosis” (English). However, the comparison is more important for languages that have much less Web content, such as Bahasa Indonesia and Turkish. Indonesia, which had the highest number of reported cases of human H5N1 viral infections in 2006 [12], had a 15-fold higher query rate for “avian flu” in Bahasa Indonesia (“flu burung”) than in English. We found that Turks used Turkish to search Google for health terms at 3- to over 1000-fold higher rates than English, French, German, or Spanish (Table 2; Multimedia Appendix 2). We did, however, find sites in Turkish that had translated information from the WHO. Whether or not the WHO, CDC, and FAO wish to leave it up to others to translate their information accurately and rapidly must be decided based on their confidence level of the eHealth capabilities of each target nation. Given the Google language-based search patterns, we conclude that during times of infectious disease outbreaks, though English may be useful, global agency–affiliated Web pages translated into local languages would likely be highly accessed and would have the benefits of being viewed as authoritative and accurate and of being transmitted in real time.

### Language of Online Mental Health Information Searches

In terms of mental health, many developing nations have 10- to 100-fold fewer psychiatrists per capita than many developed nations [17] (Table 2). For this reason, global accessibility of online mental health information has the potential to be very beneficial to physicians and families of patients in developing nations [24]. Given ethnic taboos [24], we first asked whether or not Internet users from developing nations are searching online for mental health information—potentially useful information for global mental health experts. Indeed, we found that the search rate for “schizophrenia” was similar between developed and developing nations in the local language, demonstrating an active need for online mental health information in poor countries. We have made the full mental health dataset available (Multimedia Appendix 2). Given that Google estimates 3-fold more search results concerning schizophrenia in English versus the next 10 languages combined (data not shown), we asked whether middle- and low-income nations searched for this topic in English. We found that people from Brazil, a nation of about 180 million people, searched for “schizophrenia” in Portuguese at a 28-fold higher rate than in English (Table 2). 

**Table 2:** Google search rates for selected health terms in local languages relative to English*

**Search Term**	**Country**	**Public Health Comparison Metric**	**Local Language**	**English Searches (% of Local Language Searches)**
**Avian flu**		**Human Cases^†^ (Poultry Outbreaks)^‡^**		
	United States	0 (0)	English	100.0
	France	0 (1)	French	1.6
	Germany	0 (1)	German	0.8
	Turkey	12 (176)	Turkish	0.9
	Democratic Republic of Congo	0 (0)	French	1.1
	Cote d’Ivoire	0 (3)	French	1.4
	Burkina Faso	0 (4)	French	100.0
	Mozambique	0 (0)	Portuguese	143.8
	Mexico	0 (0)	Spanish	3.3
	Brazil	0 (0)	Portuguese	1.0
	Indonesia	56 (211)	Bahasa Indonesia	6.5
**Tuberculosis**		**TB Cases**^§^		
	United States	10510	English	100.0
	France	5901	French	9.8
	Germany	5243	German	14.3
	Turkey	32371	Turkish	29.1
	Democratic Republic of Congo	307554	French	23.6
	Cote d’Ivoire	116349	French	8.6
	Burkina Faso	46815	French	15.9
	Mozambique	123360	Portuguese	28.4
	Mexico	45710	Spanish	same term
	Brazil	141115	Portuguese	5.6
	Indonesia	605759	Bahasa Indonesia	1779.3
**Schizophrenia**		**Psychiatrists per 100000^||^ People**		
	United States	13.70	English	100.0
	France	22.00	French	6.6
	Turkey	1.00	Turkish	10.5
	Democratic Republic of Congo	0.04	French	43.0
	Cote d’Ivoire	0.20	French	7.1
	Burkina Faso	0.05	French	9.5
	Mozambique	0.04	Portuguese	24.4
	Mexico	2.70	Spanish	4.1
	Brazil	4.80	Portuguese	3.6
	Indonesia	0.21	Bahasa Indonesia	115.4

^*^Based on sampling 20% of all searches on Google.com from January 2004 to April 2006.

^†^Subtype H5N1, from the WHO Epidemic and Pandemic Alert and Response Database [12], updated August 9, 2006.

^‡^Type H5 outbreaks since 2003, from the OiE [13], updated August 16, 2006.

^§^Estimated TB prevalence in 2004 from the WHO Global Tuberculosis Database [15].

^||^2005 data from Project Atlas: Resources for Mental Health and Neurological Disorders, from the WHO Global Health Atlas Database [17].

In contrast, people from Indonesia, a nation of 220 million people, searched for “schizophrenia” at similar rates in Bahasa Indonesia as in English. As with infectious disease searching, our data would suggest that users from most nations tend to search Google for “schizophrenia” in their official language at up to 10-fold higher rates than other languages (Multimedia Appendix 2). Many of the world’s people might therefore benefit if the world’s most authoritative mental health agencies (eg, US National Institute of Mental Health) translated information into other languages, even though this is not part of their domestic mandate.

### The Online Search Rates of Immigrant Minorities

In some developed nations, there is concern that immigrant groups might spread infectious diseases. In Europe, the TB prevalence is 43/100000 people in Turkey but lower in wealthier nations such as Germany (6/100000); in Asia, the TB prevalence is 273/100000 people in Indonesia, compared to 48/100000 in Singapore [15] (Table 2). Unlike many of their neighbors, both Turkey and Indonesia have reported human cases of avian influenza [12]. Governments may be interested to know whether their immigrant communities consult infectious disease–related websites originating from their adopted country or their native country. As a case study, we examined the search rates of Turks and Indonesians after they migrated to other nations in Europe or Asia, respectively, as determined by searches in Turkish and Bahasa Indonesia originating outside Turkey and Indonesia. The Turkish and Indonesian languages are distinct relative to many of their surrounding nations, permitting us to discern the search behavior of these populations after they have emigrated, provided they search in their native language. As shown in Table 3, we detected searches in Turkish for “avian flu” and “tuberculosis” throughout Europe, including high rates in Belgium and Austria, respectively. In Asia, we could detect searches in Bahasa Indonesia for “tuberculosis” and very high rates for “avian flu” throughout Asia and the Pacific region, including Hong Kong, Singapore, and Australia. We cannot exclude that these (presumed) Turkish and Indonesian immigrants also searched online in their adopted language(s), nor could we measure the fraction of their searches in their native language versus adopted language(s). However, based on the fraction of a country’s population that belongs to a particular immigrant group versus the fraction of searches conducted in the immigrant language compared to the adopted language, then if every person in a country searched the Internet for the same term at the same rate, but in different languages, we could then extrapolate that Turkish immigrants in Belgium searched for “avian flu,” “schizophrenia,” and “tuberculosis” 100%, 40%, and 15% of the time, respectively, in Turkish rather than French (Table 3). Using the same simplistic assumptions, in Austria, 39% of searches for “tuberculosis” by Turkish immigrants were in Turkish rather than German. In reality, immigrant groups are likely searching for a term such as “avian flu” at a higher rate than the general population when the corresponding disease affects their homeland and is in the news. However, we also found high search rates for “tuberculosis” and even “schizophrenia” in Turkish in these nations, which are less featured in the news. We conclude that it is important for health officials to be aware that if they wish to disseminate health information to susceptible immigrant groups, they should not rely on websites published in the majority language(s) of the nation. High priority domestic health-related websites should be multilingual, particularly those that concern infectious disease.

Our analysis also revealed surprises: for example, in Bangladesh, one of the world’s poorest and most populated nations, with 136 million residents, we found that the search rate for “avian flu” in Bahasa Indonesia (“flu burung”) was equivalent to the search rate in English (Table 3); the Bahasa Indonesia translation of “schizophrenia” was also high in Bangladesh. The high Bahasa Indonesia search rate may reflect the fact that many Bangladeshi citizens work in Singapore and Malaysia, nations that speak a similar language, Malay (Bahasa Malayu) [25]; it would appear that when they return to Bangladesh, Bangladeshis continue to use the terminology they learned while away, but the reason is unclear. We suggest that international health organizations aiding Bangladesh should publish or meta tag health information in Malay (Bahasa Malayu) to reach health practitioners in that nation. This result also highlights the practical value of analyzing linguistic preferences during online searching.

Finally, we were surprised to find that online searches for “avian flu,” “schizophrenia,” and “maize/corn” in Spanish in the United States occurred at less than 1% of the English search rate (Table 3). The US online Hispanic population (legal and illegal) is estimated to be 12% of the total US online population, of which nearly half use Spanish for some (28%) or all (21%) of their Internet usage [8,26]. Therefore, our data suggest that Latin American immigrant groups in the United States search for health information to a lesser degree in Spanish than might be predicted, although these data could also be a result of the digital divide between the groups.

**Table 3:** Online search rates of immigrant minorities

**Search Term**	**Country**	**Immigrant Language**	Comparative Major Language^*^ of Adopted Country	**Immigrant Language Searches per 10000 Major Language Searches**	**Relevant Immigrants per 10000 Total Population^†^**
**Turkish immigrants in Europe**					
Avian flu	Belgium	Turkish	French	56.1	39.7^‡^
	Switzerland	Turkish	German	9.2	N/A
	United Kingdom	Turkish	English	31.2	N/A
Tuberculosis	Austria	Turkish	German	60.2	155.4^§^
	Belgium	Turkish	French	5.8	39.7^‡^
	Germany	Turkish	German	11.8	212.2^||^
	Switzerland	Turkish	German	10.7	N/A
	United Kingdom	Turkish	English	2.8	N/A
Schizophrenia	Belgium	Turkish	French	15.9	39.7^‡^
	Switzerland	Turkish	French	62.2	N/A
	United Kingdom	Turkish	English	2.9	N/A
**Indonesian/Malaysian immigrants in Asia/Pacific**					
Avian flu	Australia	Bahasa Indonesia	English	234.7	625.2^¶^
	Bangladesh	Bahasa Indonesia	English	10474.0	N/A
	Hong Kong	Bahasa Indonesia	English	3424.4	N/A
	India	Bahasa Indonesia	English	154.4	N/A
	Singapore	Bahasa Indonesia	English	2268.0	N/A
Tuberculosis	Hong Kong	Bahasa Indonesia	English	23.3	N/A
	Singapore	Bahasa Indonesia	English	17.0	N/A
Schizophrenia	Australia	Bahasa Indonesia	English	6.0	625.2^¶^
	Bangladesh	Bahasa Indonesia	English	687.4	N/A
	Hong Kong	Bahasa Indonesia	English	295.1	N/A
	India	Bahasa Indonesia	English	16.0	N/A
**Spanish immigrants in English-Speaking North America^#^**					
Avian flu	Unites States	Spanish	English	102.8	> 503.0^**^
Schizophrenia	Unites States	Spanish Portuguese	English	91.8	> 503.0^**^
Corn (maize)^‡‡^	Unites States	Spanish Indigenous (“choclo”/”elote”)	English	37.9	> 503.0^**^

^*^a major language for which Google search rates were accessible, not necessarily the largest linguistic group of the nation.

^†^Total population data are from United Nations Population Division, 2005 data. URL: http://www.un.org/esa/population/unpop.htm.

^‡^Data are from Institut National De Statistique, 2003 data. Population et Ménages: Mouvement de la population et migrations, "Immigrations extérieures par nationalité et groupe d'âges – Belgique." URL: http://www.statbel.fgov.be.

^§^Data are from Statistics Austria, Volkszählung. Hauptergebnisse I – Österreich, 2001 census data. URL: statistik.at/neuerscheinungen/vzaustria.shtml.

^||^Data are from Statistisches Bundesamt (Federal Statistical Office, Germany), 2006 data. URL: http://www.destatis.de/themen/e/thm_bevoelk.htm.

^¶^Data are from Australia Bureau of Statistics, Cultural and Language Diversity, 2001 Census data. URL: http://www.abs.gov.au/.

^#^“Tuberculosis” is not included as it is the same term in both English and Spanish.

^**^Data are from Department of Homeland Security Yearbook of Immigration Statistics. Legal immigrants, 2005 data. URL: uscis.gov/graphics/shared/aboutus/statistics/ybpage.htm.

^‡‡^The search term used was “corn.”

**Table 4:** Search rates in European languages in Sub-Saharan Africa

**Country**	ColonialLanguage^*^	**Minority Language**	**Minority Language Searches per 10000 Colonial Language Searches**
**Search Term: Avian Flu**			
Angola	Portuguese	English	6629
Cameroon	French	German	323
Ghana	English	French	2024
Ghana	English	German	1744
Ghana	English	Dutch	498
Kenya	English	French	2941
Mozambique	Portuguese	English	14286
Mozambique	Portuguese	French	4444
Nigeria	English	French	4235
Nigeria	English	German	897
Rwanda	French	English	1481
Senegal	French	English	732
**Search Term: Tuberculosis**			
Angola	Portuguese French	English Spanish	1504
Cameroon	French Portuguese	English	2405
Democratic Republic of Congo	French Portuguese	English Spanish	2364
Ghana	English	French Portuguese	836
Kenya	English	French Portuguese	1128
Mozambique	Portuguese French	English Spanish	2842
Nigeria	English	French Portuguese	506
Rwanda	French Portuguese	English Spanish	2808
South Africa	English	Afrikaans	1547
**Search Term: Schizophrenia**			
Angola	Portuguese Spanish	English	1547
Cameroon	French	English	1922
Democratic Republic of Congo	French	English	4304
Ghana	English	French	488
Mozambique	Portuguese Spanish	English	2441
Rwanda	French	English	18453
Senegal	French	English	3287

^*^When two languages are noted, the term is the same in both languages.

### Search Rates in European Languages in Sub-Saharan Africa

Sub-Saharan Africa suffers from high rates of infectious disease, including TB and HIV [18], and high rates of malnutrition [19]. As demonstrated in Table 4, our search results suggest that the dissemination of online health or food security information to this region by international agencies should not be limited to the colonial language(s) of the target nations. For example, in English-speaking Ghana, 20% of searches for avian flu were in French (“grippe aviaire”), 17% in German (“vogelgrippe”), and 5% in Dutch (“vogelgriep”) relative to English (Table 4). In Mozambique, a former Portuguese colony, we found 1.4 times more searches for “avian flu” in English relative to Portuguese, and a high rate in French. In the Democratic Republic of Congo (DRC), a former French colony with more than 300000 TB infections in 2004 [15], 24% of the searches for tuberculosis were in English relative to French. Because Internet use is only 1.9% is Ghana, 0.7% in Mozambique, and 0.2% in DRC [8], it is plausible that these high rates of non-colonial language searches using Google may reflect searches by health professionals trained in other nations, including workers from international agencies who would be expected to have better Internet access than the general population.

### The Effect of Region-Specific Cultural and Indigenous Terminology

Finally, we measured the effects of cultural bias within the same linguistic group, using a term important for human nutrition and food security, “maize.” Maize is eaten directly, but it is also a major source of animal feed worldwide. Maize is known as “corn” in the United States and the United Kingdom, but as “maize” in many other English-speaking nations. We found that the US searches for “corn” were at a 28-fold higher rate than for “maize,” while other English-speaking nations such as Nigeria and Zimbabwe queried “maize” at a 1.5- to 4-fold higher rate than “corn,” respectively (Table 5); in the latter nations, “corn” may also refer to any large cereal grain (eg, wheat). This search behavior has consequences, as we found only three domain names that overlapped between searches for “maize” versus “corn” out of the first 50 unique Google search results.

Therefore, when African nations attempt to retrieve information about growing maize in English, they may unknowingly be excluding authoritative information from the United States and other Western English-speaking nations, such as practical information from the US National Corn Growers’ Association, whose website does not appear in the first 100 Google hits for “maize,” but ranks sixth when “corn” is searched. Similarly, when international organizations such as the FAO wish to transmit knowledge, for example, using Spanish or Portuguese in Latin America, indigenous terminology usage may make some of this information inaccessible. Maize originated from Southern Mexico and fed indigenous Latin American civilizations [27]. In Mexico and Guatemala, we found that an indigenous synonym for maize, “elote,” was searched at a similar rate to the Spanish term, “maíz” (Table 5). In Peru, however, we found that a different indigenous term, “choclo,” was searched at a 2-fold higher rate than “maíz.” We conclude that cultural and indigenous linguistic divisions may be preventing large numbers of food security and nutrition websites from reaching those people aiding 800 malnourished people or 1.2 billion agricultural workers that live in developing countries [19]. Cultural and indigenous bias may be particularly prevalent for terms related to crops, diseases, or pathogens that have pre-colonial origins.

**Table 5:** The effect of region-specific cultural and indigenous terminology

**Country**	Calories from Maize (kcal/person/day)^*^	**Search Term Comparison**	**Search Rate Ratio**
Canada	N/A^†^	corn:maize	36:1
United States	512	corn:maize	28:1
United Kingdom	115	corn:maize	7:1
India	38	corn:maize	2:1
Nigeria	179	corn:maize	1:2
Kenya	775	corn:maize	1:3
Tanzania	646	corn:maize	1:4
Zimbabwe	720	corn:maize	1:4
Spain	N/A^†^	maíz:elote:choclo	10:1:4
Venezuela	467	maíz:elote:choclo	16:1:3
Colombia	312	maíz:elote:choclo	20:1:5
Mexico	1081	maíz:elote:choclo	14:16:1
Guatemala	869	maíz:elote:choclo	12:10:1
Peru	145	maíz:elote:choclo	11:1:25
Argentina	132	maíz:elote:choclo	41:1:51

^*^Data from FAOSTAT [19].

^†^N/A: Data not available.

## Discussion

### Principal Results

In a world where infectious disease pandemics and threats of famine are always present, and in spite of the fact that the World Wide Web offers great hope for rapid and accurate sharing of information between peoples, we have demonstrated that one linguistic group does not or cannot access the health and food security websites of a different linguistic group. Our data suggest at least three reasons for this.

The first reason is that the websites of most institutions are not published in more than one or two languages. When we sampled Web pages found by the English-language queries “avian flu,” “tuberculosis,” “schizophrenia,” and “maize/corn” and their counterpart queries in other languages, the Google search results typically overlapped by only less than 10% in terms of the domain names retrieved, indicating that 90% of the relevant Web pages had not been translated into at least two languages (see Table 1). For example, when Turkish or Bahasa Indonesia was used as the search language, Web pages from very authoritative sources, such as the CDC or WHO, were not retrieved by Google. We also found that a single linguistic accent or special character in the search query could significantly alter the number and content of health-related search results retrieved by Google. Therefore, one reason for the linguistic digital divide is that the majority of health and food Web pages are not translated into multiple languages and/or that their cross-language retrieval by search engines is poor.

The first problem would not be important if the world’s online community, better educated than the general public, searched in English, since the majority of the world’s Web pages are published in English [8,9]. However, we found that there was a 2- to 100-fold higher Google search rate for health and food terms in the native language of a country compared to English (see Table 2).

Finally, within a nation, it might be assumed that language would not be a problem if a domestic agency only published their health websites in the majority language of their own people. However, we found that in Asia and Western Europe, a subset of immigrants from Indonesia and Turkey, respectively, searched Google for health and food information in their native language, not the language(s) of their adopted countries (see Table 3). In Sub-Saharan Africa, we detected unexpectedly high search rates for health information in non-colonial European languages (see Table 4), perhaps reflecting the presence of international aid workers. Finally, in Latin America, we found that indigenous words were used to search Google for information about food, rather than the colonial language of Spanish (see Table 5). Therefore, domestic agencies, in addition to global agencies, face a linguistic challenge when publishing information online: their target audiences still require information to be published in different languages, even though Internet users are presumably more educated and thus more multilingual than the general population.

### Recommendations

Given our observation that the world’s peoples appear to be searching for health and food terms in their local language or mother tongue, not in English, previous online language translation efforts by the WHO and FAO have no doubt been worthwhile. This is also revealed by the high page rank (first page results) of WHO-affiliated search results when Google is searched for infectious disease information in one of the WHO’s six online languages. We recommend that these efforts be continued and further expanded to include more languages; this recommendation applies to global agencies, but also to domestic agencies, in order to meet immigrant or indigenous needs and/or to make information accessible to other nations. To achieve such translation goals, better health-specific translation software must be developed and more translators are needed who specialize in human health and food security terminology. For example, improved cross-language search retrieval [23] of health information by online search engines would be beneficial. These investments should then be used to initially target health and food security information to the world’s most important linguistic groups, which include speakers of Chinese (1080 million people), Hindi (about 500 million), English (350-500 million), Spanish (390 million), Arabic (255 million), Portuguese (190 million), Bengali (215 million), Russian (255 million), Bahasa Indonesia (200 million), Japanese (127 million), Punjabi (104 million), German (123 million), and French (119 million) [10]. In a world that is primarily non-English speaking, such attempts will help to reduce the linguistic digital divide in health and food information on the World Wide Web.

Furthermore, as we did in this study (see Table 2 to Table 5), we recommend that when global or domestic health and food security organizations wish to use the Internet to disseminate information to other nations [28] or to their own immigrant or indigenous communities, they should first consult search engine query rates for different translations of possible search terms in order to determine which online languages are most needed. Multimedia Appendix 2 contains extensive linguistic online search pattern data to help health and food agencies better select languages for targeted website publishing. In order to measure search rates for other subjects of interest, we note that free online tools exist, such as Google Trends. In some situations, such as targeting indigenous groups, who often speak the majority language of a nation, all that may be needed is to imbed translated keywords into a majority-language website (eg, Spanish) so that search engines such as Google can cross-retrieve relevant information.

### Future Studies

Though this study examined the extent to which agencies such as the WHO are publishing information in multiple languages, we did not systematically address the quality of health and food information available in different languages. Quality analysis would especially be important for minor languages that have little content available on the Web: for example, we estimate that there are more than 4000-fold fewer search results in Bahasa Indonesia than in English for “tuberculosis,” more than 200-fold fewer search results for “avian flu” in Arabic or Japanese than in English, and about 500-fold fewer search results for “schizophrenia” in Arabic than in English. Though these precise numbers are considered to be unreliable [29], they do illustrate the point that most of the world’s linguistic groups, even major ones, have much less available online health information relative to English. As to the quality of the websites that are available, this will require systematic analysis, which poses significant methodological problems [30]. One could, however, perform a subjective survey-based evaluation by multilingual physicians, as has been performed to evaluate disease-specific websites published in English [31]. Such a survey should include quantifying to what extent online information from the WHO, CDC, and FAO is being translated into the world’s minor languages in order to help these agencies determine where they need to target their online translation efforts.

## Multimedia Appendix 1

Language translations used in this study (pdf)
					

## Multimedia Appendix 2

Rate at which users from 227 different countries searched Google (xls)
					

## Abbreviations

CDC: Centers for Disease Control
DRC: Democratic Republic of Congo
FAO: Food and Agricultural Organization
MDG: Millennium Development Goal
OiE: Organisation Mondiale de la Santé Animale [World Organisation for Animal Health]
TB: tuberculosis
WHO: World Health Organization
