# Supplementary material for: Language Preferences on Websites and in Google Searches for Human Health and Food Information
Source: J Med Internet Res. 2007 Jun 28;9(2):e18. doi: 10.2196/jmir.9.2.e18 (PMC1913940; doi:10.2196/jmir.9.2.e18)
Supplement: Supplementary file 1 [file jmir_v9i2e18_app1.pdf]

Multimedia Appendix 1. Language translations of search terms used in this study.

Language	Translation
**Tuberculosis**	
English, Spanish	tuberculosis
French, Portuguese	tuberculose
German, Danish, Afrikaans	tuberkulose
Turkish	tüberküloz
Bahasa Indonesian	tuberkulosa
**Schizophrenia**	
English	schizophrenia
French	schizophrénie
Spanish/Portuguese	esquizofrenia
Turkish	*Sizofreni
Bahasa Indonesia/Malayu	skizofrenia
**Avian flu**	
English	Avian flu
French	grippe aviaire
Spanish	gripe aviar
Portuguese	gripe aviaria, gripe aviária
German	vogelgrippe
Dutch	vogelgriep
Bahasa Indonesia	flu burung
Turkish	*Kus gribi
**Maize**	
English	maize
English	corn
Spanish	maíz
Indigenous Latin America	elote
Indigneous Latin America	choclo
Bahasa Indonesia	jagung

*Denotes the use of additional special characters.
